# Combinatorial characterization of a certain class of words and a conjectured connection with general subclasses of phylogenetic tree-child networks

**DOI:** 10.1038/s41598-021-01166-w

**Published:** 2021-11-08

**Authors:** Miquel Pons, Josep Batle

**Affiliations:** Departament de Física, Universitat de les Illes Balears, 07122 Palma de Mallorca, Balearic Islands Spain

**Keywords:** Phylogeny, Applied mathematics

## Abstract

The combinatorial study of phylogenetic networks has attracted much attention in recent times. In particular, one class of them, the so-called *tree-child networks*, are becoming the most prominent ones. However, their combinatorial properties are largely unknown. In this paper we address the problem of exactly counting them. We conjecture a relationship with the cardinality of a certain class of words. By solving the counting problem for the words, and on the basis of the conjecture, several simple recurrence formulas for general cases arise. Moreover, a precise asymptotic analysis is provided. Our results coincide with all current formulas in the literature for particular subclasses of *tree-child networks*, as well as with numerical results obtained for small networks. We expect that the study of the relationship between the newly defined words and the networks will lead to further combinatoric characterizations of this class of phylogenetic networks.

## Introduction

Evolutionary histories of several kinds are usually represented with the mathematical help of phylogenetic trees. Linguistics, but mostly genomics are the traditional areas where this tool is employed. However, mechanisms of reticulate evolution, such as horizontal gene hybridization, transfer or recombination, render such trees less appropriate. When the species involved in those events have more than one ancestor, *phylogenetic networks* are better suited^1–4^. Also, the comparison of phylogenetic trees and networks is attracting considerable attention^5,6^. Quite recently, a phylogenetic network of SARS-CoV-2 genomes was sampled from across the world in order to better understand the outbreak of the ongoing Covid-19 coronavirus world pandemic^7^.

Due to the increasing usage of phylogenetic networks, a combinatorial approach to their study regarding counting, enumeration and stochastic characterization has received a lot of attention recently^8–17^. It is usual to impose further restrictions to the general structure of phylogenetic networks (in general they are *labeled directed acyclic graphs*) in order to make them more manageable. Among all classes of phylogenetic networks, we shall study tree-child networks (TCNs)^18^, see section “Preliminaries” for a formal definition. This class, besides of being biologically justified, is considered to possess good and interesting mathematical properties, but they are considered to be largely unknown from the combinatorial point of view^17^. The present work tries to shed light on the long-sought problem of counting and enumerating this class of networks.

There exists in the literature exact counting results for TCNs with low number, *k*, of reticulation events, and arbitrary number, *n*, of leaves. Specifically for $k=1,2$ and 3, see Refs.^8,11,14^ respectively, and for TCNs with the maximum number of reticulation nodes^13^. Our conjectured formula, Eq. (13), coincides with all these results, and it also reproduces particular values obtained by demanding computational procedures^8,14^.

Specifically, we shall continue the work of Fuchs et al.^13^ who made use of a similarity from the number of maximally reticulated tree-child networks (we will show it occurs whenever $k=n-1$) with a certain class of words defined in the *On-Line Encyclopedia of Integer Sequences*, specifically the sequence OEIS A213863. They are simply related by a *n*! factor. We have discovered that all general subclasses ${\mathcal {TC}}_{n,k}$ seem to be similarly related, now by a *falling factorial* factor, to a generalized version of those words.

In fact, as commented by Flajolet and Sedgewick^19^, p. 62, words can, at least in principle, encode any combinatorial structure. A classical example is the encoding of set partitions. How to encode them can be found in the same reference, where the translation makes possible an easy counting of the set partitions. A kind of set partition called *binary total partition*, which consists in repeatedly dividing the blocks of an original set into exactly two blocks, until only singletons remain, is bijectively related to the class of binary labeled trees, that is, *phylogenetic trees* (see example 5.2.6 in Ref.^20^).

The outline of this work goes as follows. We start with a preliminary section, providing general and basic terminology and elementary properties, firstly of general phylogenetic networks and secondly concerning the class of tree-child networks. In section “Tree-child networks and words” we highlight the parallel between TCNs and words. We start by reproducing the bijection given by Fuchs et al.^13^ for the maximally reticulated subclass. In the second part of the section we establish a bijection between phylogenetic trees and a class of very similar words. To the best of our knowledge, the encoding induced by the bijection is a brand new one. We highlight some practical aspects of the encoding and we also provide a simple algorithm to generate the entire sequence of words with a minimum difference between any two consecutive ones, which is useful for exhaustive combinatorial studies of phylogenetic trees. Finally the generalized words are defined, and the relationship between them and arbitrary subclasses of TCNs is conjectured. In section “Implications of the conjecture”, and based on the conjecture, counting formulas for TCNs are derived, enumeration procedures are provided and an asymptotic formula is obtained.

## Preliminaries

In this section the basic terminology is introduced, including the formal definitions of *phylogenetic networks* and the *tree-child networks class*. Elementary, although important properties of these structures are also provided.

### Phylogenetic networks

A *phylogenetic network* ${\mathcal {N}}$ on *X* is a rooted acyclic digraph with no edges in parallel satisfying the following properties:

1. the root has in-degree zero and out-degree one.
2. a vertex with out-degree zero has in-degree one and it is called a *leaf*. The set of leaves are bijectively labeled with the elements of *X*.
3. all other vertices either have in-degree one and out-degree two, or in-degree two and out degree-one.

The vertices with in-degree two and out-degree one are called *reticulations*, and the vertices with in-degree one and out-degree two are called *tree vertices*. The edges directed into a reticulation are *reticulation edges*, and all other edges are *tree edges*. In particular, a phylogenetic *X*-tree is a phylogenetic network on *X* with no reticulations.

#### Lemma 1

*For any phylogenetic network with n leaves, k reticulation nodes and t tree nodes the following relation holds:*

1 $$\begin{aligned} n+k=t+1. \end{aligned}$$

#### Proof

The sum of the out-degrees is equal to the sum of the in-degrees. $\square$

If *u* is a vertex of a phylogenetic network ${\mathcal {N}}$ and (*u*, *v*) is an edge in ${\mathcal {N}}$, we say *v* is a *child* of *u*, conversely, *u* is a *parent* of *v*. More generally, *u* is an *ancestor* of a vertex *w* if there is a directed path from *u* to *w* in ${\mathcal {N}}$, in which case, *w* is a *descendant* of *u*.

Let ${\mathcal {R}}({\mathcal {N}})$ denote the set of reticulation nodes of the phylogenetic network ${\mathcal {N}}$. Then, let ${\mathcal {N}} - {\mathcal {R}}({\mathcal {N}})$ be the subnetwork that is obtained from ${\mathcal {N}}$ by removing all incident edges to the reticulation nodes and *edge-contract* the resulting nodes with in-degree one and out-degree one. This subnetwork is actually a *forest* in which each connected component consists only of tree nodes and it is rooted at either the network root or a former reticulation node. Each of these connected components is a *tree-component* of ${\mathcal {N}}$. This is a useful concept for characterizing the topological structures of phylogenetic networks^21,22^.

### Tree-child networks

**Figure 1:**
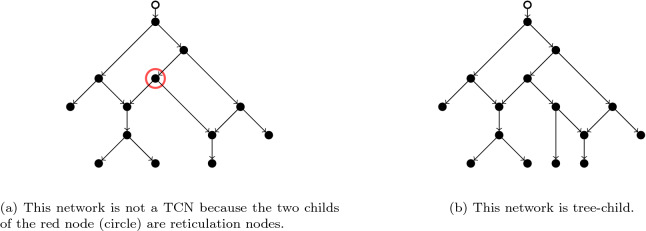
Examples of phylogenetic networks.

A phylogenetic network ${\mathcal {N}}$ on *X* is a *tree-child network* if each non-leaf vertex *v* of ${\mathcal {N}}$ has a child that is either a tree vertex or a leaf. Introduced in Ref.^18^, the class of tree-child networks is an increasingly prominent class of phylogenetic networks found in the literature. See Fig. 1 for some examples. From the definition it follows that no reticulation of ${\mathcal {N}}$ has a child reticulation and no tree vertex of ${\mathcal {N}}$ has two child reticulations. It will be convenient to define a *deep tree path* as a path starting with a node and ending with a leaf, such that all the intermediate nodes of the path are tree nodes. In a TCN there exists at least one such *deep tree path* for every node.

It is also said that a tree node is *free* if each of its children is either a tree node or a leaf. Moreover, an edge to a child of a free tree node is known as a *free edge*. In the present work we will denote the class of all tree-child networks with *n* leaves by ${\mathcal {TC}}_{n}$, their subclasses having *n* leaves and *k* reticulation nodes by ${\mathcal {TC}}_{n,k}$.

#### Lemma 2

^13^ *Every tree-child network in* ${\mathcal {TC}}_{n,k}$ *has* $n-k-1$ *free tree nodes and thus* $2(n-k-1)$ *free edges.*

#### Proof

From (1), we have that a tree-child network from ${\mathcal {TC}}_{n,k}$ has $n+k-1$ tree nodes. The two parents of every reticulation node are not free, and due to the tree-child property, different reticulation nodes have different parents. Thus, the number of tree nodes which are not free is 2*k*, from which the result follows. $\square$

#### Corollary 1

*The number of edges ending either in a tree node or a leaf, called tree edges, is equal to* $2n+k-1$.

#### Proof

Add up all contributions: one edge from the root, *k* edges from the reticulation nodes, the $2(n-k-1)$ free edges and 2*k* edges from the 2*k* that are not free. $\square$

## Tree-child networks and words

In this section we present bijections between words and two particular subclasses of tree-child networks. The first considered subclass is the set of *unlabeled* maximally reticulated TCNs. The mapping, to certain words having every letter repeated exactly three times, was given by Fuchs et al.^13^. The second case is the subclass of networks without reticulations, that is, the set of proper phylogenetic trees, with labeled leaves. We provide a bijection between phylogenetic trees and a similar class of words, but now every letter is repeated twice. In both situations the word is determined from the network by drawing and labeling non overlapping *deep tree paths*. We will see that for the maximally reticulated TCNs paths do not depend on the label of the leaves, whereas for trees, words strongly depend on the labeling of the leaves. Besides, for trees the mapping is a one-to-one relationship, whereas for the maximally reticulated TCN, every labeling of the leaves gives rise to a different TCN, hence there are exactly *n*! networks sharing the same word, being *n* the number of leaves. We finalize the section by presenting a driving hypothesis: a general TCN, say with *k* reticulation nodes and *n* leaves, is a mixture of the above cases, where the associated words have *k* letters repeated thrice and the remaining characters are repeated twice.

### Maximally reticulated tree-child networks

We start by introducing the first proposition which gives the fundamental characterization of this particular subclass of TCNs: for every node in every maximally reticulated TCN there exists an unique *deep tree path*. The property is of paramount importance in order to establish a bijection with words.

#### Proposition 1

^13^ *A tree-child network from* ${\mathcal {TC}}_{n}$ *has* $n-1$ *reticulation nodes if and only if the path from every node to a leaf whose intermediate nodes are all tree nodes is unique.*

#### Proof

First, let us assume that we have a tree-child network with *n* leaves and $n-1$ reticulation nodes. Then, for different reticulation nodes, the paths from these nodes to leaves with all intermediate nodes being tree nodes end with different leaves. Moreover, the child of the root (which is a tree node) also has a path with all intermediate nodes being tree nodes that end with another leaf. Thus, we have already at least *n* leaves and, consequently, no node can have two paths with the claimed property because the number of leaves would thus exceed *n*.

Next, let us suppose that for every node there is a unique path to a leaf with all intermediate nodes being tree nodes. Consider first this path from the child of the root. Clearly, all intermediate nodes must be parents of reticulation nodes for otherwise an intermediate node would have two different paths to leaves with all intermediate nodes being tree nodes. Moreover, any reticulation node which is the child of an intermediate node on the path is followed by a tree node, which again has a path to a leaf (all intermediate nodes being parents of reticulation nodes). Clearly, this gives a network with *n* leaves and exactly $n-1$ reticulation nodes. $\square$

Next the class of words is formally defined.

#### Definition 1

Let ${\mathcal {A}}_n$ denote the class of words built from a *n*-ary alphabet so that in each word *w* every letter is repeated exactly 3 times, and for every prefix *z* of *w* we have $\#(z,a_i) = 0$ or $\#(z,a_i) \ge \#(z,a_j)$ for all $j>i$ and the function $\#(z,a_i)$ counts the occurrences of the *i*-th letter in *z*.

The sequence $\{x_n\}_{n\ge 0} = \{1,1,7,106,2575,\ldots \}$ corresponds to the entry A213863 of the OEIS. Bellow the first classes are shown:

$$\begin{aligned} {\mathcal {A}}_0&= \emptyset \\ {\mathcal {A}}_1&= \{aaa\}\\ {\mathcal {A}}_2&= \{aabbab, ababab, baabab, aaabbb, aababb, abaabb, baaabb\} \end{aligned}$$

The following result was recently discovered by Fuchs et al.^13^. Note that for maximally reticulated TNC the *tree-components*, defined in section “Phylogenetic networks”, are actually paths, so in this section we will refer to them as *path-components*.

#### Proposition 2

*There is a bijection from the set of tree-child networks* ${\mathcal {TC}}_{n,n-1}$ *with labels removed to* ${\mathcal {A}}_{n-1}$. *Consequently,* $\vert {\mathcal {A}}_{n-1}\vert = \vert {\mathcal {TC}}_{n,k} \vert / n!$.

#### Proof

Beginning with any given network from ${\mathcal {TC}}_{n,n-1}$, the bijection goes as follows.

In the first step, we sort the path-components of the chosen tree-child network. We do this inductively. First, the path-component of the child of the root receives an index 0. Assume that *k* path-components have been indexed. Now, consider all un-indexed path-components whose first node (which is a reticulation node) has its two parents already within indexed path-components. If both parents are in the same path-component, then one is the descendant of the other. Let us call that one the *second parent*. If both parents are in different path-components, then the parent in the path-component with the higher index is the *second parent*. Now, sort all the above chosen un-indexed path-components according to the indices of the path-components where the second parents are located. In case their indices coincide, one goes to the ancestor relationship within the path-component of their second parents. Continue this until all path-components are indexed, which will eventually happen because our networks are assumed to be connected.

Now, we label the first node of every path-component of index $k>0$ together with its two parents by $a_k$.

**Figure 2:**
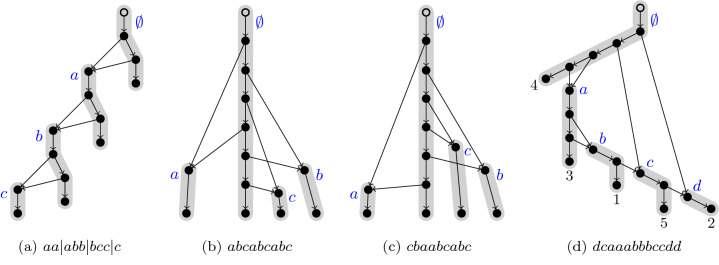
Some encodings of *Maximally Reticulated Tree-Child Networks*. In panel (**a**) bars have been used to explicitly delimitate the path-components. As explained in the Proof of Proposition 2, the bars can be discarded without losing information.

Finally, we read the labels of each path-component starting with the 0th one until reaching the last one; see Fig. 2, where a line separates the strings from different path-components, although they are not necessary (they ever occur just before the third appearance of a letter).

The resulting word uses $n-1$ letters, $a_1,\ldots ,a_{n-1}$ with each letter repeated exactly thrice. Moreover, if a letter of the resulting word when read from the left occurs from the first time, then due to the above construction, no larger letter could have occurred already twice. Likewise, if a letter occurs from the second time, again no larger letter could have occurred already thrice. Therefore, the resulting word satisfies the property from Definition 1.

Since the construction does not depend on the labels of the leaves, and clearly it can be reversed, the resulting map constitutes a bijection between *unlabeled* maximally reticulated TCNs and words.

The construction actually establishes a total strict order on the set of leaves, determined by the name of the paths themselves. Thus there are exactly n! (labeled) networks in ${\mathcal {TC}}_{n,n-1}$ associated to the same word.

$$\square$$

Fuchs et al.^13^ made use of these words as an auxiliary tool to determine the cardinality of the ${\mathcal {TC}}_{n,n-1}$ subclass, and to discern its asymptotic behavior. However, they are important in their own right because statistical properties of the words have a direct reflection on the topology of the network. Consider for instance the mean distance between equal letters and their dispersion. Next some results about *Means* and *Standard Deviations* are provided. The proofs can be found in the section A of the Supplementary Information File (SIF), along with the proofs of the analogous results presented in the next section.

The lowest possible mean value is zero and it corresponds to the word $aaabbbccc\ldots$, the associated network, Fig. 2a, is very regular. The maximum mean value is equal to $n-1$ and is achieved by words of the type $a_{\sigma (1)} a_{\sigma (2)} \ldots a_{\sigma (n)} a\, b\, c \ldots a_n a\, b\, c \ldots a_n$, where $\sigma$ is any permutation of $\{1,2,\ldots ,n\}$. Taking into account that in such words the distance between the first and second appearances is $x(i)=n-1+\sigma (i)-i$, the *Standard Deviation* (SD) is given by

$$\begin{aligned} {\mathrm {SD}} = \sqrt{\sum _{i=1}^n \frac{(x(i)-{\bar{x}})^2+(n-1-{\bar{x}})^2}{2n}}=\sqrt{\frac{1}{2n}\sum _{i=1}^n (\sigma (i)-i)^2}. \end{aligned}$$

It ranges from zero for the identity permutation, to a maximum value of $\sqrt{(n^2-1)/6}$, corresponding to the permutation $(n, n-1,\ldots ,1)$. These two cases correspond to Fig. 2b,c, respectively. Words having the maximum SD are of the form $zy\,\,\ldots \,\,rq\,aaabbb\,\,\ldots \,\,ppp\,qqrr\,\,\ldots \,\,zz$, where *p* is the letter located in the $n-s$ alphabet’s position and *z* refers to the last character of whatever *n*-ary alphabet. The length *s* of the prefix $zy\,\,\ldots \,\,q$ yielding a maximum SD depends on *n*, going asymptotically to the value $s=(2-\sqrt{2})\left( n - \frac{1}{4} + {\mathcal {O}}\left( \frac{1}{n}\right) \right)$ and implying a linear increase of the maximum SD. The concomitant value is $\frac{1}{2}\sqrt{21-12\sqrt{2}}\,\left( n-\frac{6-3\sqrt{2}}{28-16\sqrt{2}}+{\mathcal {O}}\left( \frac{1}{n}\right) \right)$, and they have a mean value of $\frac{3}{2}(\sqrt{2}-1)\,n - \frac{3}{8}\sqrt{2} + {\mathcal {O}}\left( \frac{1}{n}\right)$. For $n=4$ the specific word is *dcaaabbbccdd*, and the highly irregular TCN associated to it is shown in Fig. 2d.

### Phylogenetic trees

Now we move to the other extreme case: TCNs with no reticulation nodes, that is, *phylogenetic trees*. Next we show that there is a bijective relationship between phylogenetic trees with *n* leaves and words over an alphabet of $n-1$ letters satisfying conditions similar to those of Definition 1. In this case letters are now repeated exactly twice instead of thrice. This is stated in the following proposition:

#### Proposition 3

*The set of phylogenetic trees on *[*n*] *taxa* *is bijectively related to the class of words* ${\mathcal {B}}_{n-1}$ *built from a* $(n-1)$*-ary alphabet, so that in each word w every letter is repeated exactly twice, and for every prefix z of w we have* $\#(z,a_i) = 0$ *or* $\#(z,a_i) \ge \#(z,a_j)$ *for all* $j>i$, *and the function* $\#(z,a_i)$ *counts the occurrences of the i-th letter in z.*

#### Proof

We shall give the bijection. Suppose that the leaves are labeled with elements of $\{1,2,\ldots ,n\}$. First, let us make the assignments $\{2 \rightarrow a, 3 \rightarrow b, 4 \rightarrow c, \ldots \}$. Next, let us successively index *deep paths* in the following way: assign index 0 to the path from the root to the leaf labeled with number 1. For every node of the path, different from the root and the leaf, consider the path from the child of that node (not belonging to the path) to the descendant leaf with the lowest label. Then, index that path according the label of the leaf and the former assignment. Since any two members of the (root) path can not have common descendants not belonging to the path, it does not matter the order in which the indexing is done.

Continue then until the entire tree is covered by *deep paths*, which will eventually happen because our trees are assumed to be connected.

**Figure 3:**
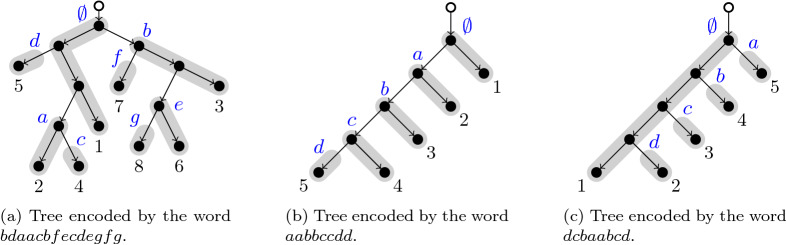
Examples of Tree encodings.

Finally, we read the labels of each indexed path, starting with the 0th one, continue with the *a* path, writing firstly the name of the path followed by the name of all paths departing from it. Then proceed lexicographically until reaching the last one (see Fig. 3 for some examples).

The resulting word uses $n-1$ letters, $a_1,\ldots ,a_{n-1}$ with each letter is repeated exactly twice. Moreover, if a letter of the resulting word, when read from the left occurs from the first time, then due to the above construction, no larger letter could have occurred already twice.

Finally, it is straightforward to see that the above construction can be reversed. Thus, the resulting map is a bijection. $\square$

Next the first sets ${\mathcal {B}}_n$ are displayed.

$$\begin{aligned} {\mathcal {B}}_0 &= \emptyset \quad \text {(The empty word.)} \\ {\mathcal {B}}_1&= \{aa\}. \\ {\mathcal {B}}_2&= \{aabb, abab, baab\}. \\ {\mathcal {B}}_3&= \{aabbcc, ababcc, baabcc, aabcbc, abacbc, baacbc, aacbbc,\\&\quad abcabc, bacabc, acabbc, acbabc, bcaabc, caabbc, cababc, cbaabc\}. \end{aligned}$$

Let us now determine the cardinalities $\vert {\mathcal {B}}_n\vert$. Given the set corresponding to an alphabet of $n-1$ letters, in order to obtain the set associated with *n* letters one shall add to each word two repetitions for the new letter (the last one in the alphabet). One of them must necessarily go at the end, and the other one may go elsewhere, thus having $2n-1$ possibilities. Consequently $|{\mathcal {B}}_n|=(2n-1)|{\mathcal {B}}_{n-1}|=(2n-1)!!$.

The bijection provides an easy encoding of phylogenetic trees, making their comparison straightforward. However, the codification is not a *succinct representation* because it uses twice the number of strictly necessary bits. This fact is seen by considering the leading term in the asymptotic expansion of the number $(2n-3)!!$ of phylogenetic trees, which is $\sqrt{2}\left( \frac{2n-2}{e}\right) ^{n-1}$. The number of bits (obtained by taking the base 2 logarithm of the previous expression) goes as $(n-1)\log _2(n-1)$. On the other hand, our words contain $2(n-1)$ letters, and for each letter one shall require $\log _2(n-1)$ bits. Still, the proposed encoding is not much larger than Newick’s standard codification, which only uses *n* numbers but it also requires $2n-2$ parenthesis and $n-1$ commas. The important fact here is that Newick’s encoding is not a bijection.

As in the case of maximally reticulated networks, statistical analysis of the words can be performed. The very important difference is that in the former case words did not depend on the labels of the leaves, thus they only affect the topology of the network. But for phylogenetic trees, words depend not only on the topology but also on the labeling. Popular statistical indices for trees, such as the Colless or Sackin indices do not depend on the labeling, reflecting the structure of the tree. Therefore, taking into account the names of the leaves in the statistical indices can only be useful to compare trees with the same names for the leaves, or to be significative if some non arbitrary or significant order can be established between the set of leaves, which represent the so-called *extant species*. Next some statistical properties of the words are stated. The reader is referred to the section A of the SIF for their proofs. Regarding as before the distance between equal letters , words with the minimum average distance (actually zero) $aabbcc\ldots$ correspond to trees with lowest labels located as close to the root as possible, see Fig. 3b. On the other hand, words with the largest mean distance, specifically $n-1$, correspond as before to trees with the maximal depth, but now with the lowest label located as far as possible to the root. They are all words of the form $a_{\sigma (1)} a_{\sigma (2)} \ldots a_{\sigma (n)} a\, b\, c \ldots a_n$, where $\sigma$ is any permutation of $\{1,2,\ldots ,n\}$, and the variance ranges from zero for the identity permutation, to a maximum value of $(n^2-1)/3$ reached when the permutation is $(n, n-1,\ldots ,1)$. This latter case is represented in Fig. 3c. Words having the maximum variance are of the form $zy\,\,\ldots \,\,rq\,aabb\,\,\ldots \,\,pp\,qr\,\,\ldots \,\,yz$, where we assumed that *z* is the *n*th letter of the alphabet and *p* is the letter located at the $(n-s)$th position of the alphabet. The length *s* of the prefix $zy\,\,\ldots \,\,q$ yielding a maximum SD depends on *n*, going asymptotically to the value $s=\left( \frac{3-\sqrt{5}}{2}\right) \,n + \frac{2\sqrt{5}-5}{10} + {\mathcal {O}}\left( \frac{1}{n}\right)$. This fact implies a linear increase of the maximum Standard Deviation, specifically $\text {SD} \propto \frac{5-\sqrt{5}}{2\sqrt{3}}\,\left( n-\frac{3}{5}+{\mathcal {O}}\Bigl (\frac{1}{n}\Bigr )\right)$, having a mean value of $\left( \frac{\sqrt{5}-1}{2}\right) \,n - \frac{\sqrt{5}}{5} + {\mathcal {O}}\left( \frac{1}{n}\right)$.

As remarked by Diaconis and Holmes^23^, combinatorialists often seek ways of walking through the space of all objects in a step-by-step way, useful for example to evaluate phylogenetic algorithms. This goal can be achieved by generating all equivalent words with minimal changes. The following program does this job. For convenience letters $a,b,c\ldots$ are substituted by numbers $1,2,3\ldots$

**Algorithm T** (*Gray tree generation*): This algorithm visits all 2*n*-words $w=c_1 c_2 \ldots c_{2n}$ that satisfy the conditions stated in Proposition 3 by starting with the word $112233\ldots nn$ and doing minimal changes at a time, until overflow occurs. Variable *i* contains the character to be moved next and an auxiliary vector of parities $d_2 d_3 \cdots d_n$ is used to reproduce the mirror symmetry.

**T1. [Initialize.]**: Set $c_{2j-1}\leftarrow j$ and $c_{2j}\leftarrow j$ for $1\le j \le n$, and set $d_j\leftarrow 0$ for $2\le j \le n$. Also set $i\leftarrow 2$ (, the first letter to be moved).
**T2. [Visit.]**: Visit the word $w=c_1 c_2 \ldots c_{2n}$. (The program that wants to examine all 2*n*-words now does its thing.)
**T3. [Locate char to move.]**: Let $j\ge 1$ be minimum such that $c_j=i$. Set $a\leftarrow c_j$.
**T4. [Direction?]**: If $d_i=0$ go to T5, otherwise ($d_i=1$) go to T6.
**T5. [Move left.]**: Let $k<j$ be maximum such that $c_k<c_j$. If $k>0$ go to 7. On the contrary, set $d_i\leftarrow 1$ and $i\leftarrow i+1$. Terminate if $i=n+1$, otherwise go back to T3.
**T6. [Move right.]**: Let $k>j$ be minimum such that $c_k\le c_j$. If $c_k\ne c_j$ go to 7. On the contrary, set $d_i\leftarrow 0$, $i\leftarrow i+1$ and go back to T3.
**T7. [Exchange.]**: Set $c_j \leftarrow c_k$ and $c_k \leftarrow a$. Also set $i \leftarrow 2$ and go to T2.

The output sequence for $n=3$ (corresponding to trees with 4 leaves) is

$$\begin{array}{llllll}1 &1 &2 &2 &3 &3\\ 1& 2 &1 &2 &3 &3\\ 2 &1 &1 &2 &3 &3\\ 2 &1 &1 &3 &2 &3\\ 1 &2& 1 &3 &2 &3\end{array} \qquad \begin{array}{llllll}1& 1& 2& 3& 2& 3\\ 1& 1& 3& 2& 2& 3\\ 1& 2& 3& 1& 2& 3\\ 2& 1& 3& 1& 2& 3\\ 2& 3& 1& 1& 2& 3\end{array}\qquad \begin{array}{llllll}1& 3& 2& 1& 2& 3\\ 1& 3& 1& 2& 2& 3\\ 3& 1& 1& 2& 2& 3\\ 3& 1& 2& 1& 2& 3\\ 3& 2& 1& 1& 2& 3\end{array}$$

### The conjecture

In this section we present a conjecture about the number of general subclasses of tree-child networks over *n* taxa and the cardinality of the set of words in the alphabet $\{a_1, a_2, \ldots , a_{n-1}\}$ that satisfy similar properties as the ones specified in Definition 1 and Proposition 3. Firstly the class of words is formally defined.

#### Definition 2

Let ${\mathcal {C}}_{n,k}$ denote the class of words built from a *n*-ary alphabet, where all words have the same length, $2n+k$. Every word *w* has *k* letters repeated thrice, $n-k$ letters occur twice and for every prefix *z* of *w* we have $\#(z,a_i) = 0$ or $\#(z,a_i) \ge \#(z,a_j)$ for all $j>i$. The function $\#(z,a_i)$ counts the occurrences of the *i*-th letter in *z*.

Sets ${\mathcal {A}}_n$ and ${\mathcal {B}}_n$ are included in this definition. Next we display the class ${\mathcal {C}}_{2,1}$ as a minimal proper example of the general case.

$$\begin{aligned} {\mathcal {C}}_{2,1}= & {} \{aabba, ababa, baaba, aaabb, aabab, abaab, baaab\}. \end{aligned}$$

Section B of the SIF contains a bigger example: the ${\mathcal {C}}_{3,1}$ class. The main conjecture simply proposes an specific connection between cardinalities.

#### Conjecture 1

The cardinality of a (general) subclass ${\mathcal {TC}}_{n,k}$ of tree-child networks with *n* leaves and *k* reticulation nodes is related to the cardinality of the class of words ${\mathcal {C}}_{n-1,k}$ by the equality

2 $$\begin{aligned} \vert {\mathcal {TC}}_{n,k} \vert = \frac{n!}{(n-k)!}\,\,\times \,\,\vert {\mathcal {C}}_{n-1,k}\vert . \end{aligned}$$

The conjecture is supported by strong evidence. By solving the counting problem concerning ${\mathcal {C}}_{n,k}$, the numbers of tree-child networks predicted by the hypothesis (2) exactly coincide with all entries of the table provided by Cardona and Zhang^8^ which cover all cardinalities of TCN subclasses up to eight leaves. Furthermore simple analytic expressions can be extracted from the hypothesis, which coincide with already proven formulas for low numbers of reticulation nodes deduced by different methods: case $k=1$ solved by L. Zhang in Ref.^14^, case $k=2$ proved by Cardona and Zhang^8^ and the case $k=3$ provided by Fuchs et al.^11^. Of course it also coincides with the extreme cases $k=0$ and $k=n-1$. Incidentally, due to a theorem provided in Ref.^8^, it also agrees with the case $k=n-2$.

Once Conjecture 1 is proven, which can be done by proving from properties of tree-child networks any of the forthcoming results based on it (namely Propositions 8, 9, 10 or 11), any surjective map

$$\begin{aligned} \psi \ : \ {\mathcal {TC}}_{n,k}\, \longrightarrow \, {\mathcal {C}}_{n-1,k}\ , \end{aligned}$$

will induce a *partition* on the set ${\mathcal {TC}}_{n,k}$ through the *equivalence relation*

$$\begin{aligned} \text {Given } x,y \in {\mathcal {TC}}_{n,k}:\quad x\sim y\ \Leftrightarrow \ \psi (x)=\psi (y). \end{aligned}$$

The map will be crucial to relate the stochastic and combinatorial properties of words with those properties of tree-child networks. Certainly the task should be simpler if the map produced a partition with equal-sized blocks. There is no evidence at this point indicating that it shall be the case. However, since this is the more desirable scenario, we consider that such “*natural map*” is worth searching for.

#### Conjecture 2

*There exists a surjective map* $\psi :\, {\mathcal {TC}}_{n,k} \longrightarrow {\mathcal {C}}_{n-1,k}$ *such that all its equivalence classes* $[a]=\{x \in {\mathcal {TC}}_{n,k} : \psi (a)=\psi (x)\}$ *have the same size, that is,*

$$\begin{aligned} \#\,[a] = \frac{n!}{(n-k)!}\quad \forall \; a\,\in \, {\mathcal {TC}}_{n,k}. \end{aligned}$$

Another ambitious objective would be to enlarge somehow the words in such a way that it could be possible to define a bijection between ${\mathcal {TC}}_{n,k}$ and a new class ${\mathcal {C}}^{\textstyle *}_{n-1,k}$ of “*augmented words*”. It will make possible a convenient encoding of TCNs, facilitating their comparison. For the very particular case of maximally reticulated TCNs, the extension is immediate: one only needs to add the label of the final leaf of every *deep path* after the third appearance of the corresponding letter. In this fashion, the tree-child network represented in Fig. 2d is encoded as *dcaaa*3*bbb*1*cc*5*dd*2.

## Counting words

In this section several counting formulas for the classes of words ${\mathcal {C}}_{n,k}$ are derived, as well as a precise asymptotic result. Next proposition is somehow fundamental.

### Proposition 4

*The number of words specified in Definition* 2, $c_{n,k} \equiv \vert {\mathcal {C}}_{n,k}\vert$, *satisfies the recurrence*

3 $$\begin{aligned} c_{n,k}=c_{n,k-1} + (2n+k-1)\,\,c_{n-1,k} \qquad \text {with} \ c_{0,0}=1 \ \hbox {and} \ c_{i,-1}=c_{i,i+1}=0 \,\ \forall \ i. \end{aligned}$$

### Proof

Notice that for every word $w \in {\mathcal {C}}_{n,k}$ the *k* letters repeated three times need to be the first *k* letters of the alphabet, otherwise the entire word will not fulfill the prefix condition. For the same reason, each word $w \in {\mathcal {C}}_{n,k}$ either ends with the *k*th letter or with the *n*th one.

In the first case, the map consisting in adding the *k*th letter at the end of a word $w \in {\mathcal {C}}_{n,k-1}$ determines a one-to-one correspondence between ${\mathcal {C}}_{n,k-1}$ and the subset $X \subseteq {\mathcal {C}}_{n,k}$ of words ending with $a_k$: Adding $a_k$ to two different words $w_1\ne w_2\, \in {\mathcal {C}}_{n,k-1}$ produces two different words $w'_1\ne w'_2\, \in X$, and due to the prefix condition removing the last letter of a word $w' \in X$ leads to a word belonging to ${\mathcal {C}}_{n,k-1}$. It accounts for the first term on the right hand side of Eq. (3).

Words comprised in the second case can be obtained by adding to each word $w \in {\mathcal {C}}_{n-1,k}$ two repetitions of the *n*th letter. Since one letter is located at the end of the word, the other letter can be placed in any of the $2n+k-1$ available positions. It accounts for the second term on the right hand side of Eq. (3).

Incidentally, in case $k=n$, the fist procedure, which places the last $n{\mathrm {th}}-$ letter at the end of each word $w \in {\mathcal {C}}_{n,n-1}$, already generates all words with three repetitions, hence the convenience to define $c_{i,i+1}=0$.

Finally, for the other extreme case $k=0$, adding a third appearance of a letter does not apply, so it is imposed $c_{i,-1}=0$, obtaining the recurrence for the double factorials $c_{n,0}=(2n-1)!!$. $\square$

The lowest values of the cardinalities $c_{n,k}$ are:

4 $$\begin{array}{c} 1\\ 1\quad 1\\ 3\quad 7\quad 7\\ 15\quad 57\quad 106\quad 106\\ 105\quad 561\quad 1515\quad 2575\quad 2575\\ 945\quad 6555\quad 23220\quad 54120\quad 87595\quad 87595\\ \end{array}$$

where rows and columns correspond to *n* and *k*, respectively.

### Proposition 5

*The numbers* $c_{n,k}$ *satisfy the following recurrence equations*

5a $$\begin{aligned} c_{n+1,k}= \ \sum _{r=0}^k\, (2n+r+1)\,c_{n,r}, \end{aligned}$$

5b $$\begin{aligned} c_{n,k+1}= \sum _{\ell =k+1}^n \,\prod _{i=\ell +1}^n\!(2i+k)\;c_{\ell ,k}. \end{aligned}$$

### Proof

Both equations follow by considering Eq. (3) as a first order recurrence equation, $y_{m+1}=a_m y_m + b_m$, and then applying its standard solution method, see section D of the SIF. To obtain Eq. (5a), the independent term $b_m$ must be identified with $c_{n,k-1}$, solve for *n* and after that, shift *n* by one unit. On the other hand, to obtain Eq. (5b) the independent term has to be identified with $(2n+k-1)\,\,c_{n-1,k}$, and then to solve for *k*. $\square$

**Figure 4:**
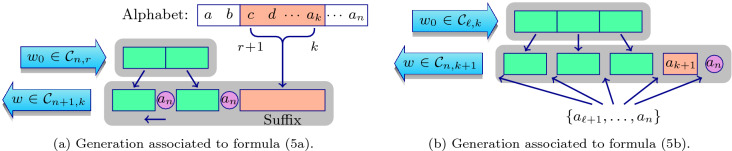
Methods to recursively generate the classes of words.

Every counting equation implicitly describes a recursive procedure to generate the entire class. Formula (5a) implies that all words composing ${\mathcal {C}}_{n+1,k}$ can be obtained by adding up disjoint sets, one for each member of the sum (5a). All words belonging to one such set share a common suffix formed by $k-r$ ordered letters of the alphabet, from the $(r+1)$-th to the *k*-th letter. Different prefixes are built from words in ${\mathcal {C}}_{n,r}$, adding the $(n+1)$-th letter of the alphabet twice, one at the end of the word and the other one inserted in all possible positions. See diagram (a) shown in Fig. 4.

Regarding the other expression, Eq. (5b), the members of an arbitrary disjoint set are obtained from words belonging to $C_{\ell ,k}$ by first adding the $(k+1)$-th letter of the alphabet at the end of that word. Then add two repetitions of the $(\ell +1)$-th letter, one at the end and the other in all possible positions. After that add similarly the $(\ell +2)$-th letter, one placed at the end and the other one elsewhere. Continue doing so until placing the last letter of the alphabet. The method is depicted in the diagram (b) displayed in Fig. 4.

### Enumeration

Given a combinatorial class, an important issue is to being able to list all its elements. In section F of the SIF we provide an algorithm, based on the recurrence (5a), that sequentially generates all words in ${\mathcal {C}}_{n,k}$. Here we describe the recursive structure on which it is based, although the algorithm is not written recursively.

Given numbers *n* and *k*, all words in ${\mathcal {C}}_{n,k}$ are generated starting by the word (written in numbers)

6 $$\begin{aligned} w_0=112233\ldots nn||123\ldots k . \end{aligned}$$

The algorithm can be understood as a sequence of “*intermediate initial configurations*”. One such “*intermediate*” word has the structure

7 $$\begin{aligned} w_i=p(n\!-\!1)\,|\,q_1(i)\,|\,nn\,||\,q_2(i) \end{aligned}$$

where the substring $p(n\!-\!1)$ is precisely $1122\ldots n\!-\!1\,\,n\!-\!1$, while substrings $q_1$ and $q_2$ are the two parts of a cut of the rightmost *k* numbers in the original word (6), thus $q_1(i)=12\ldots i\!-\!1$ and $q_2(i)=i\,\,i\!+\!1\ldots k$. The sequence starts with $i=0\ (q_1=\emptyset )$ and ends with $i=k$. From this basis word, other words are obtained by moving the left most *n* to the left, placing it in all possible positions. But every one of such movements will be done after the same procedure is applied to the (inner) word

8 $$\begin{aligned} w'=p(n\!-\!1)\,||\,q_1(i). \end{aligned}$$

In the Section B of the SF it can be seen how the above procedure generates all words in ${\mathcal {C}}_{3,1}$.

Thus we take an *enumeration* of ${\mathcal {C}}_{n,k}$, that is, a bijective mapping from ${\mathcal {C}}_{n,k}$ to an initial segment of the natural numbers, as the position in which the last procedure visits a word. Due to the clear recursive structure of the procedure, it is both quick and simple to implement, given an arbitrary word in which position it is visited. The following algorithm does this job.

**Algorithm E** (*Direct Enumeration*): Given an input word $w=c_1 c_2 \ldots c_m$, this algorithm determines the class $C_{n,k}$ to which it belongs and equipped with the table of cardinalities $c_{n,k}$ (Proposition 4), it provides the position *P* that Algorithm A (placed in Section F of the SIF) visits it.

**E1. [Initialize.]**: Determine *n*, the number of distinct characters. Also determine *k*, the number of characters repeated thrice. Initialize the output $P\leftarrow 1$, the position of the word in the list.
**E2. [Easy case?]**: If $k=n$ remove rightmost character (it is necessarily $a_n$). Set $k\leftarrow k-1$.
**E3. [Localize last char.]**: Find *p* and $q<p$ maximums such that $c_p=c_q=a_n$.
**E4. [Actualize.]**: Remove last $2n+k-p$ characters and the other repetition of $a_n$ located at position *q*. Set $k\leftarrow p-2n$ and also set $n\leftarrow n-1$.
**E5. [Accumulate.]**: Set $P \leftarrow P \,+\, (p-q-1)\,c_{n,k}\,+\,\sum _{r=0}^{k-1}(2n+r+1)\,c_{n,r}$. Terminate if $n=0$, otherwise go to E2.

It is of course possible to reverse the last algorithm. That is, specifying *n*, *k* and an integer $1 \le P \le c_{n,k}$, to determine to which word it corresponds. Such algorithm could be useful to generate words uniformly at random.

### Asymptotic behavior

Let us start by providing an useful counting formula for small values of *k*.

#### Proposition 6

*The number of words contained in* ${\mathcal {C}}_{n,k}$ *is given by*

9 $$\begin{aligned} c_{n,k}=\sum _{i=0}^k \frac{(2n+2k-i-1)!!}{(k-i)!}\,a_i , \end{aligned}$$

*where the coefficients* $a_i$ *are determined by the recursive equation*

10 $$\begin{aligned} a_i = - \sum _{j=1}^i \frac{(3i-3+j)!!}{(3i-3)!!}\,\frac{a_{i-j}}{j!}, \quad \text { and } \ a_0=1. \end{aligned}$$

*The first terms of the* $a_i$ *sequence are* $\{1, -1, \frac{1}{6}, \frac{17}{48}, -\frac{283}{1512}, -\frac{467}{9216}, \frac{66329}{1297296}, \frac{8915}{4644864}, \ldots \}$.

The proof follows easily by induction. It can be found in the Section C of the SIF.

Formula (9) is very appropriate to study the asymptotic behavior for *k* fixed and $n \rightarrow \infty$. Notice that the variable *n* only appears in the argument of the double factorial, thus for sufficiently large *n* only considering the first term of the sum will be a good approximation.

#### Proposition 7

*For k fixed and* $n\rightarrow \infty$, *numbers* $c_{n,k}$ *grow as*

11 $$\begin{aligned} c_{n,k}&= \sqrt{2}\,\frac{e^{-n}\,(2n)^{n+k}}{k!}\Biggl \lbrace 1 - \sqrt{\frac{\pi }{2}}\,k\,(2n)^{-1/2} +\frac{14k^2-2k-1}{12}\,(2n)^{-1} - \sqrt{\frac{\pi }{2}}\,k\,\frac{31k^2+3k-26}{48}\,(2n)^{-3/2} \\&\quad +\frac{2900k^4-264k^3-10016k^2+6876k+21}{6048}\,(2n)^{-2} + {\mathcal {O}} \left( n\right) ^{-5/2}\Biggr \rbrace \end{aligned}$$

The detailed proof can be found in the Section E of the SIF.

It is possible to get an idea of what it means “for sufficiently large *n*”. Formula (9) will give a good asymptotic result whenever the terms of the sum are decreasing in module. Considering the known inequality between double factorials

$$\begin{aligned} m+\frac{1}{4}< \frac{(2m)!!^2}{\pi (2m-1)!!^2}< m+\frac{1}{2}, \end{aligned}$$

it can be seen that an absolute ratio of consecutive terms lower than one is obtained if

12 $$\begin{aligned} n \,>\, (k-i)^2\,\,\gamma _i \,-\,k\,+\,\frac{2i+1}{4} \quad \forall \ \; 0 \le i \le k, \end{aligned}$$

where

$$\begin{aligned} \gamma _i=\frac{1}{\pi }\left( \frac{a_{i+1}}{a_i}\right) ^2. \end{aligned}$$

Numerical experiments showed us that $\gamma _i$ is usually small but has some peaks. For $i<500$ it is ever lower than 30. It seems reasonable to consider it as a constant. In such case a safety bound would be around $n>{\mathcal {O}} \left( k^2\right)$.

## Implications of the conjecture

It is plain that it would be desirable to establish a “*natural mapping*”, as the one described in the Conjecture 2, between the subclass of tree-child networks ${\mathcal {TC}}_{n,k}$ and the class of words ${\mathcal {C}}_{n,k}$, or even better, a bijective relationship between ${\mathcal {TC}}_{n,k}$ and some class ${\mathcal {C}}^{\textstyle *}_{n, k}$ (for now unknown) of “*augmented words*”. Such bijection would provide a proper codification for the networks that would greatly help in their comparison, the enumeration procedures provided in section 4.1 could be used, but it would specially help to study the combinatorial and stochastic properties of those networks, linking them to the properties of words. Summing up, the bijection would greatly help to characterize a “typical network”. But usually it is not so simple to design a bijection. It is often much simpler to count directly the elements of a set rather than to provide a bijection with a different set whose counting is known. In order to fully prove our Conjecture 1, Eq. (2), it may be easier to do so probably via some inductive argument, by relating the cardinals of subclasses of networks. Thus, given the main Conjecture 1 and the deduced recurrence between words, Proposition 4, we state the following proposition:

### Proposition 8

*Assuming Conjecture* 1*holds, by Proposition* 4, *the cardinalities* $\vert {\mathcal {TC}}_{n,k} \vert$ *satisfy*

13 $$\begin{aligned} (n-k)\,\,\vert {\mathcal {TC}}_{n,k} \vert = (n+1-k)(n-k)\,\,\vert {\mathcal {TC}}_{n,k-1} \vert \, +\, n\,\,(2n+k-3)\,\,\vert {\mathcal {TC}}_{n-1,k} \vert , \end{aligned}$$

*with initial values* $\vert {\mathcal {TC}}_{1,0} \vert = 1$  *and*  $\vert {\mathcal {TC}}_{i,-1}\vert =\vert {\mathcal {TC}}_{i,i}\vert =0 \,\ \forall \ i$.

This recurrence exactly reproduces the table given in Ref.^8^. Table1 displays the (conjectured) counts of TCNs up to 10 leaves and all possible reticulation numbers.

**Table 1:** Counts of TCNs with *k* reticulations on [*n*], where $1 \le k < n$ and $2 \le n \le 10$.

*k*\*n*	2	3	4	5	6	7	8	9	10
1	2	21	228	2805	39,330	623,385	11,055,240	217,237,545	4,689,345,150
2		42	1272	30,300	696,600	16,418,430	405,755,280	10,606,551,480	294,109,704,000
3			2544	154,500	6,494,400	241,204,950	8,609,378,400	306,699,077,160	11,115,708,408,000
4				309,000	31,534,200	2,068,516,800	113,376,463,200	5,717,669,504,400	277,928,391,510,000
5					63,068,400	9,737,380,800	920,900,131,200	70,028,853,426,000	4,748,839,899,804,000
6						19,474,761,600	4,242,782,275,200	547,410,697,041,600	55,220,314,578,912,000
7							8,485,564,550,400	2,482,302,981,614,400	419,465,496,844,800,000
8								4,964,605,963,228,800	1,878,972,235,938,000,000
9									3,757,944,471,876,000,000
Total	2	63	4044	496,605	101,832,930	31,538,905,965	13,771,649,608,920	8,070,383,687,681,385	6,116,640,702,036,483,150

The last row contains the total numbers of TCNs.

Similarly, simply adding the falling factorial to Eq. (5a), the following result is obtained.

### Proposition 9

*Assuming Conjecture* 1*holds, the following relation, directly deduced from Eq.* (5a)*, also holds:*

14 $$\begin{aligned} (n-k)!\,\vert {\mathcal {TC}}_{n,k} \vert = n\,\sum _{r=0}^k (2n+r-3)\,(n-1-r)!\,\vert {\mathcal {TC}}_{n-1,r}\vert . \end{aligned}$$

Now, the inductive argument would consist of adding reticulations nodes starting from the initial set of phylogenetic trees with $n-1$ leaves or, alternatively, to begin with an arbitrary TCN and to start deleting reticulations until reaching a tree.

The following equality follows similarly from Eq. (5b) by adding the falling factorial.

### Proposition 10

*Assuming Conjecture* 1*holds, yet another relation for* $\vert {\mathcal {TC}}_{n,k}\vert$ *can be readily obtained from Eq.* (5b):

15 $$\begin{aligned} \vert {\mathcal {TC}}_{k+m+1,k}\vert = \sum _{\ell =0}^m\, (\ell +2) \left[\, \prod _{i=\ell +1}^m\!\left( 1+\frac{k}{i+1}\right) \Bigl (2i+3k-1\Bigr )\right]\,\vert {\mathcal {TC}}_{k+\ell +1,k-1}\vert , \end{aligned}$$

*valid for* $$k\ge 1$$.

This last Eq. (15) relates the number of elements of ${\mathcal {TC}}_{n,k}$ with those of the networks with one less reticulation, as well as all possible number of leaves. In this case the inductive reasoning would involve deleting leaves till reaching a maximally reticulated TCN, a particular subclass already counted in Refs.^11,13^. Since we believe that this is a promising way to prove the conjecture, Eq. (2), particular instances of this recurrence for low values of *m* are displayed bellow.

16a $$\begin{aligned} \vert {\mathcal {TC}}_{k+1,\,k}\vert&= 2 \,\vert {\mathcal {TC}}_{k+1,\,k-1}\vert \end{aligned}$$

16b $$\begin{aligned} \vert {\mathcal {TC}}_{k+2,\,k}\vert&= 3 \,\vert {\mathcal {TC}}_{k+2,\,k-1}\vert + \Bigl (1\!+\!\frac{k}{2}\Bigr )(3k+1)\,2\,\vert {\mathcal {TC}}_{k+1,\,k-1}\vert \end{aligned}$$

16c $$\begin{aligned} \vert {\mathcal {TC}}_{k+3,\,k}\vert&= 4 \,\vert {\mathcal {TC}}_{k+3,\,k-1}\vert + \Bigl (1\!+\!\frac{k}{3}\Bigr )(3k+3)\biggl (\!3\,\vert {\mathcal {TC}}_{k+2,\,k-1}\vert + \Bigl (1\!+\!\frac{k}{2}\Bigr )(3k+1)\,2\,\vert {\mathcal {TC}}_{k+1,\,k-1}\vert \!\biggr ) \end{aligned}$$

Let us notice how, in this fashion, we recover the principal recurrence (13):

$$\begin{aligned} \vert {\mathcal {TC}}_{k+m+1,k} \vert = (m+2)\,\,\vert {\mathcal {TC}}_{k+m+1,k-1} \vert \, +\, \Bigl (1+\frac{k}{m+1}\Bigr )\,\,(3k+2m-1)\,\,\vert {\mathcal {TC}}_{k+m,k} \vert . \end{aligned}$$

The first Eq. (16a) was already proven in Ref.^8^ (Theorem 12 therein). We presume that proving the particular case (16b) is a decisive step towards the proof of the conjecture. Moreover we think that the correct interpretation of Eq. (15) could bring us to the desired map. We believe so because the letters repeated thrice in our words are the first of the alphabet, then a feasible strategy would consist in removing leaves, possibly in an ordered way, from a given TCN until reaching a maximally reticulated TCN, then setting the labels of the paths according to the *Zhang/Yu/Fuchs bijection*, Proposition 2, and after that reconstructing the network adding the previously removed leaves.

Relation (9) provides a straightforward method for obtaining explicit formulae for the number of TCNs with few reticulations. Let us rewrite it in terms of the new coefficients $b_i\equiv i!\,a_i$.

### Proposition 11

*Assuming Conjecture* 1*holds, a convenient expression to determine* $\vert {\mathcal {TC}}_{n,k} \vert$ *for low k values is immediately deduced from Proposition* 6:

17 $$\begin{aligned} \vert {\mathcal {TC}}_{n,k} \vert = \left( {\begin{array}{c}n\\ k\end{array}}\right) \sum _{i=0}^k \left( {\begin{array}{c}k\\ i\end{array}}\right) \,(2n+2k-i-3)!!\,b_i , \end{aligned}$$

*where the coefficients* $b_i$ *are determined by the recursive equation*

18 $$\begin{aligned} b_i = - \sum _{j=1}^i \left( {\begin{array}{c}i\\ \,j\,\,\end{array}}\right) \frac{(3i-3+j)!!}{(3i-3)!!}\,b_{i-j}, \quad \text { and } \ b_0=1. \end{aligned}$$

*The first terms of the sequence are* $\{1, -1, \frac{1}{3}, \frac{17}{8}, -\frac{283}{63}, -\frac{2335}{384}, \frac{331645}{9009}, \ldots \}$. *Notice that Eq.* (18) *also follows from the combination of Eq.* (17) *with the condition* $\vert {\mathcal {TC}}_{n,n} \vert = 0$.

The number of TCNs in closed form can be obtained analytically with the help of the previous relation (17). We shall list in the following some of them:

19a $$\begin{aligned} \vert {\mathcal {TC}}_{n,1} \vert&= \left( {\begin{array}{c}n\\ 1\end{array}}\right) \biggl \lbrace (2n\!-\!1)!! - (2n\!-\!2)!!\biggr \rbrace \end{aligned}$$

19b $$\begin{aligned} \vert {\mathcal {TC}}_{n,2} \vert&= \left( {\begin{array}{c}n\\ 2\end{array}}\right) \biggl \lbrace (2n\!+\!1)!! - 2(2n)!! + \frac{1}{3}(2n\!-\!1)!!\biggr \rbrace \end{aligned}$$

19c $$\begin{aligned} \vert {\mathcal {TC}}_{n,3} \vert&= \left( {\begin{array}{c}n\\ 3\end{array}}\right) \biggl \lbrace (2n\!+\!3)!! - 3(2n\!+\!2)!! + (2n\!+\!1)!! + \frac{17}{8}(2n)!!\biggr \rbrace \end{aligned}$$

19d $$\begin{aligned} \vert {\mathcal {TC}}_{n,4} \vert&= \left( {\begin{array}{c}n\\ 4\end{array}}\right) \biggl \lbrace (2n\!+\!5)!! - 4(2n\!+\!4)!! + 2(2n\!+\!3)!!+\frac{17}{2}(2n\!+\!2)!! - \frac{283}{63}(2n\!+\!1)!! \biggr \rbrace \end{aligned}$$

Last expression, $k=4$, is actually a conjecture, whereas the previous ones agree and simplify the existing proven formulas in a compact manner. An equivalent expression to (19a) was first provided by Zhang^14^, a direct formula for TCNs with two reticulation nodes was first given by Cardona and Zhang^8^, while a closed formula for $\vert {\mathcal {TC}}_{n,3} \vert$ can be found in Ref.^11^.

**Figure 5:**
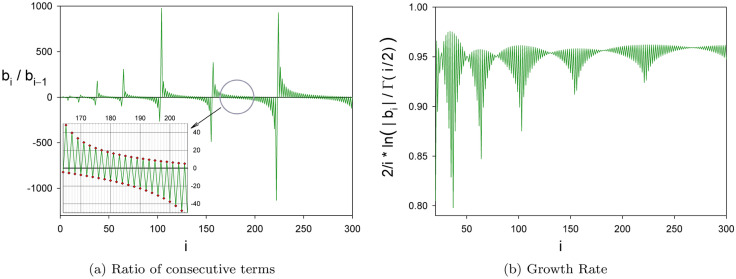
Coefficients $b_i$ follows the sign pattern $\scriptscriptstyle \cdots \,++--++--\,\cdots$ most of the time. This is reflected in a near perfect sign alternation of the ratios $b_i/b_{i-1}$, as shown in the left panel (**a**). To study the growth of coefficients $b_i$, the right panel (**b**) depicts the quantity $\frac{2}{i}\ln \!\left( \frac{\vert b_i \vert }{\Gamma (i/2)}\right)$.

Coefficients $b_i$ form a rather odd sequence, as can be grasped from Fig. 5. There it is plotted the ratio of two consecutive terms, Panel 5a. As can be seen this quantity oscillates for every increment of one unit of *i*, this means that $b_i$ coefficients can be grouped in pairs of consecutive elements having the same sign. We have checked numerically that this happens almost all the time. Also, the overall tendency of $|b_i|$ seems to be bounded by a known expression, as shown in the right panel 5b. It appears as if  $\dfrac{2}{i}\ln \!\left( \dfrac{\vert b_i \vert }{\Gamma (i/2)}\right) \,<\,1\,\,\forall \,i$.

**Figure 6:**
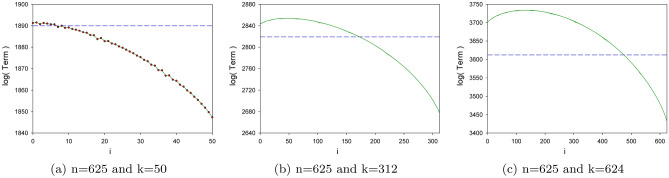
Decimal logarithm of the absolute value of the terms forming the sum (17), including the $$\left( {\begin{array}{c}n\\ k\end{array}}\right)$$ factor. Blue dashed line corresponds to the logarithm of the final sum result.

It is also instructive to study the numerical behavior of the terms composing the sum (17) for big *n* and several *k* values. In particular, in Fig. 6 we depict, for a fixed $n=625$ and several *k*, the logarithm of the absolute value of all the terms forming the sum. In each case, the horizontal line represents the logarithm of the sum result. The first panel depicts the moduli for $(n=625,k=50)$, with $k<\sqrt{n}$; the second one, $(n=625,k=312)$, with $k\approx n/2$; finally, the third one depicts $(n=625,k=624)$, that is, $k=n-1$. Bearing in mind that the first term in the sum (17) constitutes an absolute upper bound to the sum itself, great cancelations occur in the summation for each case until each curve reaches the horizontal line.

### Asymptotic expression

The concomitant expression for $\vert {\mathcal {TC}}_{n,k} \vert$ is obtained following (11) with $n \rightarrow n-1$, and adding the factor $\frac{n!}{(n-k)!}$.

#### Proposition 12

*Assuming Conjecture* 1*holds, for k fixed and* $n\rightarrow \infty$, *numbers* $\vert {\mathcal {TC}}_{n,k} \vert$ *grow as*

20 $$\begin{aligned} \vert {\mathcal {TC}}_{n,k} \vert&= \left( {\begin{array}{c}n\\ k\end{array}}\right) \sqrt{2}\,e^{-n}\,(2n)^{n-1+k}\Biggl \lbrace 1 - \sqrt{\frac{\pi }{2}}\,k\,(2n)^{-1/2} +\frac{14k^2-26k+11}{12}\,(2n)^{-1} \\&\quad - \sqrt{\frac{\pi }{2}}\,k\,\frac{31k^2-93k+70}{48}\,(2n)^{-3/2} +\frac{2900k^4-14376k^3+25264k^2-19332k+5565}{6048}\,(2n)^{-2} \\&\quad + {\mathcal {O}} \left( n^{-5/2} \right) \Biggr \rbrace \end{aligned}$$

Fuchs et al.^10^ were able to reproduce the asymptotic expressions of $\vert {\mathcal {TC}}_{n,k} \vert$ for large *n* and $k=1,2,3$ by employing a rather involved method based on generating functions. We recover the (few) previously known expansions and extend them to any (fixed) k and (sufficiently large) n, along with several additional corrections to the leading term.

**Figure 7:**
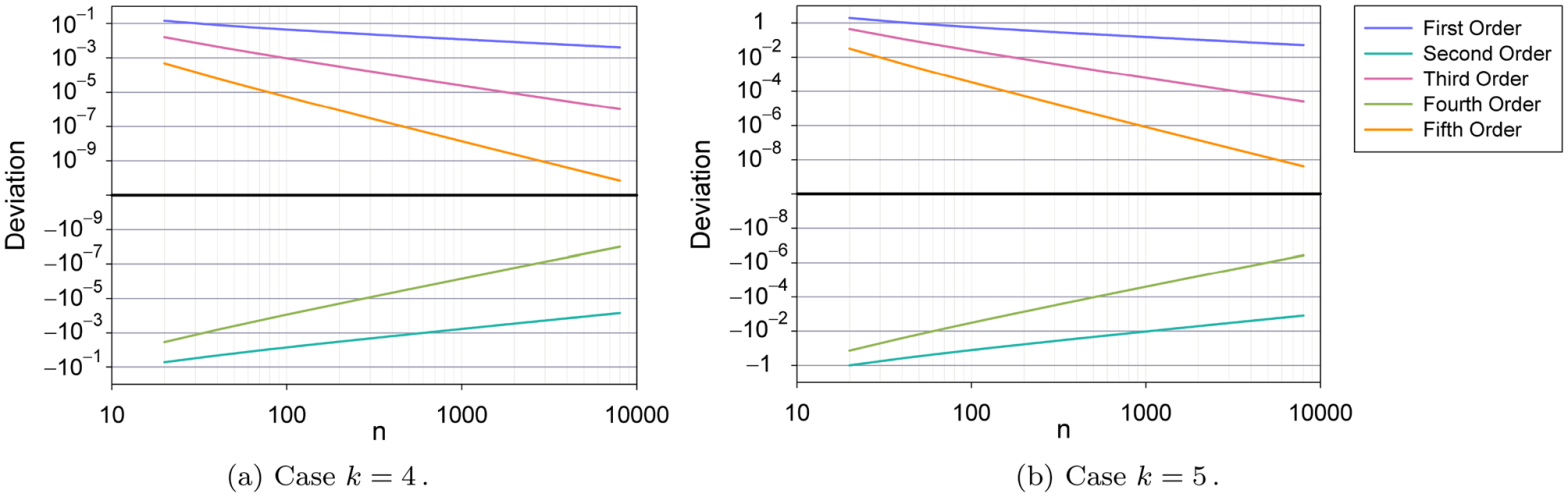
Plots of the evolution $\vert {\mathcal {TC}}_{n,k} \vert$ versus *n*, for two particular cases, $k=4$ and 5. The deviation is computed with regards to the exact value as $\text {Deviation}=\frac{\text {Approximated}}{\mathrm{Exact}}-1$.

In the Fig. 7 we depict the accuracy of the asymtotic series (20) at different orders of approximation as a function of *n* for two particular cases, $k=4 \text { and } 5$. Usually, but not always, successive approximations overestimate and underestimate the exact value.

## Concluding remarks

The key contribution deals about a combinatorial characterization of a new class of words ${\mathcal {C}}_{n,k}$, formally introduced in Definition 2. Counting formulas have been provided, enumeration procedures are described and an asymptotic analysis has been performed.

The practical importance of this class of words is a potential relationship with the subclasses ${\mathcal {TC}}_{n,k}$ of tree-child networks with *n* leaves and *k* reticulation nodes. We conjectured that the cardinalities of the classes are related by $\vert {\mathcal {TC}}_{n,k}\vert =\frac{n!}{(n-k)!}\times \vert {\mathcal {C}}_{n-1,k}\vert$.

No counterexamples have been found. Additionally, general counting formulas for ${\mathcal {TC}}_{n,k}$, deduced from the counting results on words and the conjecture, agree and simplify all already proved results for particular subclasses of tree-child networks. The present work poses a very specific challenging problem to the Phylogeny’s community: the conjecture will be proven if any of the counting formulas deduced from it (namely Propositions 9–11) can be proven only using properties of the networks.

Maps from networks to words have been described for two very particular subclasses: phylogenetic trees and maximally reticulated tree-child networks. For the trees case the map is a one-to-one correspondence, whereas for the maximally reticulated networks every word is the image of exactly *n*! distinct networks (only differing in the labels of the leaves). To dispose of a map for the general case will make possible a precise stochastic characterization of the networks. This assertion is grounded on the fact that Algorithm E can be readily reversed to sample words uniformly at random. Thus, words have the potential to characterize typical tree-child networks, namely, the typical depth, the number of linages in the ancestry of a leave, the expected lengths of random walks between the root and leaves, and so on.

Finally, we would like to address some questions. Tree-child networks seem to be related to words with letters repeated two and three times, however, the “prefix condition” can be naturally extended to words with letters repeated two, three, four,..., up to an arbitrary number of times. Therefore, could those words be related to some class of *labeled directed acyclic graphs*, as in Conjecture 1? Does it correspond to a known class of phylogenetic networks? Future work will try to elucidate the previous issues.

## Supplementary Information

Supplementary Information.
