# Supplementary material for: Combinatorial characterization of a certain class of words and a conjectured connection with general subclasses of phylogenetic tree-child networks
Source: Sci Rep. 2021 Nov 8;11:21875. doi: 10.1038/s41598-021-01166-w (PMC8575882; doi:10.1038/s41598-021-01166-w)
Supplement: Supplementary file 1 — Supplementary Information. [file 41598_2021_1166_MOESM1_ESM.pdf]

Supplementary Information

Contents

A Proofs of the statements about means and dispersions

B List of all words in $\mathcal{C}_{3,1}$

C Proof of Proposition 6

D Solution of the first order difference equation

E Proof of the asymptotic formula, Proposition 7

F Generator of a general class of words $\mathcal{C}_{n,k}$

A Proofs of the statements about means and dispersions

Proposition 1. *Words of the type* $\alpha_{\sigma(1)}\alpha_{\sigma(2)}\cdots\alpha_{\sigma(n)}a\,b\,c\cdots\alpha_n \in \mathcal{B}_n$*, where* σ *is any permutation of* $\{1,2,\ldots,n\}$*, have:*

a) *The highest mean distance, specifically* $\bar{x} = n-1$*, among all words belonging to* $\mathcal{B}_n$*.*

b) *A maximum variance equal to* $(n^2-1)/3$*, attained by the reversing order permutation:* $\sigma(i) = n+1-i$*.*

Proof. a) To maximize the distance between characters, the second repetition of a character needs to occur after all other characters have appeared. If it was not the case, i.e. there is an arbitrary letter α whose second appearance happens before all other letters have appeared once, the movement of the second appearance of α to the right, down to the left of the second appearance of any other letter that occurs first will increase the distance between the couple of α letters in m units, $m \geq 1$ by hypothesis, and the distance between the other jumped letters would be increased in one unit as well.

The "*prefix condition*" stated in Definition 2 enforces last n characters to appear in lexicographic order. Regarding the arrangement of the first n letters, note that for the identity permutation all distances are equal to $n-1$. To see that any permutation leads to the same mean distance just observe that any arbitrary movement of a single letter m positions to the right (left) decreases (increases) the distance with its counterpart in m units but at the same time each of the m "jumped over" characters increase (decrease) their distance in one unit.

b) Taking into account that the distance between characters is $x(i) = n-1+\sigma(i)-i$, and using a) we have:

$$\mathrm{Var} = \sum_{i=1}^{n} \frac{(x(i)-\bar{x})^2}{n} = \frac{1}{n}\sum_{i=1}^{n}(\sigma(i)-i)^2 = \frac{1}{3}(n+1)(2n+1) - \frac{2}{n}\sum_{i=1}^{n} i\,\sigma(i)\,.$$

As stated by the "*rearrangement inequality*", the rightmost summation is minimized by the reversing order permutation. Then

$$\mathrm{Var}_{\max} = \frac{1}{n}\sum_{i=1}^{n}(n+1-2i)^2 = \frac{1}{3}(n^2-1)$$

□

Proposition 2. *Words of the type* $zy\cdots rq\,aabb\cdots pp\,qr\cdots yz$ *have a maximal Standard Deviation among all words belonging to* $\mathcal{B}_n$*. This maximum value tends toward* $\mathrm{SD} \propto \frac{5-\sqrt{5}}{2\sqrt{3}}\left(n - \frac{3}{5} + \mathcal{O}\left(\frac{1}{n}\right)\right)$ *as* $n \to \infty$ *.*

Proof. The first part of the proof has the objective to reduce the "search space" for words with largest variance. It is proved by induction that words with largest variance have the structure shown in the enunciate. That is, with an unspecified length s of the prefix string $zy\cdots rq$, which shall determined by the end of the proof. In this fashion, the possibility $s = 0$, that never corresponds to a word with largest variance, will be ever considered.

For $n = 2$ the enunciate holds because the word with largest variance is *baab*, which belongs to the search space $\mathcal{S} = \{aabb,\, baab\}$. By the induction hypothesis, we shall assume that for every $m < n$, words with largest variance have the stated structure. Now we want to prove that this assumption implies that any potential word with length $2n$ has the same structure.

Given any word $w \in \mathcal{B}_n$, consider the initial string of m letters placed just before the second appearance of a. These m letters, conforming the set we call X, need to be distinct because the first letter (starting from the left) that can be repeated twice is a. Notice that the s rightmost letters of the word have to be the s last letters of the alphabet and they have to appear in lexicographic order. In addition to this, the second appearances (to the right of a) of the letters in X need also to appear in lexicographic order, but with the possibility to have some other letters intercalated. Then, by the *rearrangement inequality*, we know that the arrangement of the s leftmost letters in reverse lexicographic order leads to the largest variance. Let us now perform the appropriate permutation on the initial s-substring and look for the lowest k_1 ($\leq s$) such that the letter placed in the position k_1, say $w_{k_1} \equiv \alpha$ is different to $a_{n+1-k_1} \equiv \beta$. Now interchange letter w_{k_1} with the first appearance of the missing letter β, located at the position k_3, i.e. $w_{k_3} = \beta$. Next we show that this operation ever raises the variance. Since the first appearances of two letters have been interchanged, the mean distance is not modified, and thus we only need to analyze the change in the sum of squared distances. If the second appearances of letters α and β occur at positions k_2 and k_4 respectively, the change in the variance is

$$\begin{aligned} n\left(\mathrm{Var}_{\mathrm{new}} - \mathrm{Var}_{\mathrm{old}}\right) &= (k_4 - k_1 - 1)^2 + (k_2 - k_3 - 1)^2 - (k_2 - k_1 - 1)^2 - (k_4 - k_3 - 1)^2 \\ &= 2\,(k_3 - k_1)(k_4 - k_2) > 0\,. \end{aligned}$$

Therefore the change is always positive because, evidently, $k_3 > k_1$ and the "*prefix condition*" forces $k_4 > k_2$.

The following step is to perform similar swaps with the rest of the letters of the (updated) X set. The process will end with a word having an s-prefix formed by the last s letters of the alphabet placed in reverse lexicographic order, and ending with an s-suffix formed by the same last s letters, but placed in lexicographic order. No other operation can be performed on the prefix or suffix to increase the variance. Note that the central string will now belong to $\mathcal{B}_{n-s}$, then by the induction hypothesis we conclude that the overall construction has the correct structure.

The length of the initial tail $zy\cdots rq$ depends on n. To fix it, one can leave it as a parameter. Next we calculate the variance in terms of this parameter and we seek those values of s that maximize the variance.

In terms of the length s of the prefix, the mean distance of such a word with $2n$ letters is

$$\bar{x}(s) = \frac{1}{n}\sum_{i=1}^{s} 2(n-i) = 2s - \frac{s(s+1)}{n}\,. \tag{A1}$$

And the variance is given by

$$\mathrm{Var}(s) = \frac{4}{n}\sum_{i=1}^{s}(n-i)^2 - \bar{x}^2 = \frac{4s}{n^2}\left(n^3 - (2s+1)\,n^2 + \frac{1}{6}(8s^2 + 9s + 1)\,n - \frac{1}{4}s(s+1)^2\right)\,. \tag{A2}$$

Considering s to be a continuous parameter, the real function Var has a unique maximum in the interval $0 < s < n$, which can be determined by imposing the extreme condition:

$$\frac{\mathrm{d}}{\mathrm{d}s}\mathrm{Var}(s) = 0 = -\frac{4}{n^2}\left(s^3 - \left(4n - \frac{3}{2}\right)s^2 + \left(4n^2 - 3n + \frac{1}{2}\right)s - n\left(n^2 - n + \frac{1}{6}\right)\right)\,. \tag{A3}$$

By inspecting the solutions of this cubic polynomial, and taking the limit $n \to \infty$, we find that the length of the prefix scales as

$$s_{\max} = \left(\frac{3-\sqrt{5}}{2}\right) n + \frac{2\sqrt{5}-5}{10} + \mathcal{O}\left(\frac{1}{n}\right)\,. \tag{A4}$$

Substituting this value into Eqs. (A1) and (A2) yields

$$\begin{aligned}\bar{x}(s_{\max}) &\propto \left(\frac{\sqrt{5}-1}{2}\right) n - \frac{\sqrt{5}}{5} + \mathcal{O}\left(\frac{1}{n}\right) , \\ \mathrm{Var}(s_{\max}) &\propto \frac{5}{6}(3-\sqrt{5})\, n^2 - (3-\sqrt{5})\, n + \mathcal{O}(1) \ .\end{aligned}$$

And finally the Standard Deviation is determined with an standard asymptotic expansion:

$$\mathrm{SD}_{\max} = \sqrt{\mathrm{Var}(s_{\max})} \propto \frac{5-\sqrt{5}}{2\sqrt{3}} \left(n - \frac{3}{5} + \mathcal{O}\Big(\frac{1}{n}\Big)\right)$$

□

Proposition 3. *Words of the type* $a_{\sigma(1)}a_{\sigma(2)}\cdots a_{\sigma(n)}a\,b\,c\cdots a_n a\,b\,c\cdots a_n \in \mathcal{A}_n$*, where* σ *is any permutation of* $\{1,2,\ldots,n\}$*, have:*

a) *The highest mean distance, specifically* $\bar{x} = n-1$*, among all words belonging to* $\mathcal{A}_n$*.*

b) *A maximum variance equal to* $(n^2-1)/6$*, attained by the reversing order permutation:* $\sigma(i) = n+1-i$*.*

Proof. a) To maximize the distance between characters, the second repetition of a character, starting from the left, needs to occur after all other characters have appeared, as explained in the proof of Proposition 1. By the same argument the third repetition of a character needs to occur after all other characters have been appeared twice. Then, to maximize the mean distance, the letters must be grouped in three strings of n distinct letters.

To fulfill the "*prefix condition*" the second and third substrings (starting from the left) need to appear in lexicographic order. Regarding the arrangement of the first n letters, note that for the identity permutation all distances are equal to $n-1$. Clearly the same reasoning in Proposition 1 applies, and any permutation leads to the same mean distance.
b) Taking into account that the distance between the first and second appearances is $x(i) = n-1+\sigma(i)-i$, and every distance between the second and third appearance of a letter is equal to $n-1$, we have:

$$\mathrm{Var} = \sum_{i=1}^{n} \frac{(x(i)-\bar{x})^2 + (n-1-\bar{x})^2}{2n} = \frac{1}{2n}\sum_{i=1}^{n}(\sigma(i)-i)^2 = \frac{1}{6}(n+1)(2n+1) - \frac{1}{n}\sum_{i=1}^{n} i\,\sigma(i)\,.$$

As stated by the "*rearrangement inequality*", the rightmost summation is minimized by the reversing order permutation. Then

$$\mathrm{Var}_{\max} = \frac{1}{2n}\sum_{i=1}^{n}(n+1-2i)^2 = \frac{1}{6}(n^2-1)$$

□

Unfortunately we have not been able to formally prove, as in Proposition 2, that words in $\mathcal{A}_n$ with largest variance are of the form $zy\cdots rq\, aaabbb\cdots ppp\, qqrr\cdots zz$. However, it is intuitively clear that it must be so. Largest variances will be achieved in words having the largest possible distances between letters, and the least distances, actually zero. Clearly, largest distances are obtained by the following construction

$$w(a,b,\ldots,z) = z\, w'(a,b,\ldots,y)\, zz\,.$$

In this fashion, we are adding the maximum distance and the minimum one. Notice that the repetition of any letter can not be at the beginning of the word because the prefix condition would prevent the formation of any gap. Once largest distances are assured, in general the possibility to have more letters at zero distance is left open, to keep the large distances they can only correspond to groups of three repeated letters placed at the central part of the word. Again, due to the prefix condition, the repetitions need to appear in lexicographic order.

Proposition 4. *Assuming that words of the type $zy\cdots rq\, aaabbb\cdots ppp\, qqrr\cdots zz$ have a maximal Standard Deviation among all words in $\mathcal{A}_n$. The length s of the suffix $zy\cdots rq$ that maximizes the variance tends to* $(2-\sqrt{2})\left(n-\frac{1}{4}+\mathcal{O}\left(\frac{1}{n}\right)\right)$ *as* $n\longrightarrow\infty$*. Implying that the maximal Standard Deviation tends to* $\mathrm{SD}\propto\frac{1}{2}\sqrt{21-12\sqrt{2}}\left(n-\frac{6-3\sqrt{2}}{28-16\sqrt{2}}+\mathcal{O}\left(\frac{1}{n}\right)\right)$ *as* $n\to\infty$ *.*

Proof. Let us calculate the variance in terms of the length s of the initial tail $zy\cdots rq$. The mean distance of such word is

$$\bar{x}(s)=\sum_{i=1}^{s}\frac{3(n-i)}{2n}=\frac{3}{2}\,s-\frac{3\,s(s+1)}{4n}\,. \tag{A5}$$

And the variance is given by

$$\mathrm{Var}(s)=\frac{9}{2n}\sum_{i=1}^{s}(n-i)^2-\bar{x}^2=\frac{9s}{4n^2}\left(2\,n^3-(3s+2)\,n^2+\frac{1}{3}(5s^2+6s+1)\,n-\frac{1}{4}s(s+1)^2\right)\,. \tag{A6}$$

Considering s to be a continuous parameter, the real function Var has a unique maximum in the interval $0<s<n$, which can be determined by imposing the extreme condition:

$$\frac{\mathrm{d}}{\mathrm{d}s}\mathrm{Var}(s)=0=-\frac{9}{4n^2}\left(s^3-\left(5n-\frac{3}{2}\right)s^2+\left(6n^2-4n+\frac{1}{2}\right)s-n\left(2n^2-2n+\frac{1}{3}\right)\right)\,. \tag{A7}$$

By inspecting the solutions of this cubic polynomial, and taking the limit $n\to\infty$, we find that the length of the prefix scales as

$$s_{\max}=(2-\sqrt{2})\left(n-\frac{1}{4}+\mathcal{O}\left(\frac{1}{n}\right)\right) \tag{A8}$$

Substituting this value into Eqs. (A1) and (A2) yields

$$\bar{x}(s_{\max})\propto\frac{3}{2}(\sqrt{2}-1)\,n-\frac{3}{8}\sqrt{2}+\mathcal{O}\left(\frac{1}{n}\right)$$

$$\mathrm{Var}(s_{\max})\;\propto\;\frac{3}{4}\left((7-4\sqrt{2})\,n^2-(6-3\sqrt{2})\,n+\mathcal{O}\left(1\right)\right)\,.$$

And finally the Standard Deviation is determined with an standard asymptotic expansion:

$$\mathrm{SD}_{\max}\;\propto\;\frac{1}{2}\sqrt{21-12\sqrt{2}}\left(n-\frac{6-3\sqrt{2}}{28-16\sqrt{2}}+\mathcal{O}\left(\frac{1}{n}\right)\right)$$

□

B List of all words in $\mathcal{C}_{3,1}$

Next all words belonging to the class $\mathcal{C}_{3,1}$ are displayed in the same order as Algorithm A (see section F) visits them. Applying Algorithm E to any word returns the indicated ordinal number.

1:	$a\,a\,b\,b\,c\,c\,a$	20:	$a\,a\,b\,a\,b\,c\,c$	39:	$b\,a\,c\,a\,b\,a\,c$
2:	$a\,b\,a\,b\,c\,c\,a$	21:	$a\,b\,a\,a\,b\,c\,c$	40:	$a\,a\,c\,a\,b\,b\,c$
3:	$b\,a\,a\,b\,c\,c\,a$	22:	$b\,a\,a\,a\,b\,c\,c$	41:	$a\,a\,c\,b\,a\,b\,c$
4:	$a\,a\,b\,c\,b\,c\,a$	23:	$a\,a\,b\,b\,c\,a\,c$	42:	$a\,b\,c\,a\,a\,b\,c$
5:	$a\,b\,a\,c\,b\,c\,a$	24:	$a\,b\,a\,b\,c\,a\,c$	43:	$b\,a\,c\,a\,a\,b\,c$
6:	$b\,a\,a\,c\,b\,c\,a$	25:	$b\,a\,a\,b\,c\,a\,c$	44:	$a\,c\,a\,b\,b\,a\,c$
7:	$a\,a\,c\,b\,b\,c\,a$	26:	$a\,a\,a\,b\,c\,b\,c$	45:	$a\,c\,b\,a\,b\,a\,c$
8:	$a\,b\,c\,a\,b\,c\,a$	27:	$a\,a\,b\,a\,c\,b\,c$	46:	$b\,c\,a\,a\,b\,a\,c$
9:	$b\,a\,c\,a\,b\,c\,a$	28:	$a\,b\,a\,a\,c\,b\,c$	47:	$a\,c\,a\,a\,b\,b\,c$
10:	$a\,c\,a\,b\,b\,c\,a$	29:	$b\,a\,a\,a\,c\,b\,c$	48:	$a\,c\,a\,b\,a\,b\,c$
11:	$a\,c\,b\,a\,b\,c\,a$	30:	$a\,a\,b\,c\,b\,a\,c$	49:	$a\,c\,b\,a\,a\,b\,c$
12:	$b\,c\,a\,a\,b\,c\,a$	31:	$a\,b\,a\,c\,b\,a\,c$	50:	$b\,c\,a\,a\,a\,b\,c$
13:	$c\,a\,a\,b\,b\,c\,a$	32:	$b\,a\,a\,c\,b\,a\,c$	51:	$c\,a\,a\,b\,b\,a\,c$
14:	$c\,a\,b\,a\,b\,c\,a$	33:	$a\,a\,a\,c\,b\,b\,c$	52:	$c\,a\,b\,a\,b\,a\,c$
15:	$c\,b\,a\,a\,b\,c\,c$	34:	$a\,a\,b\,c\,a\,b\,c$	53:	$c\,b\,a\,a\,b\,a\,c$
16:	$a\,a\,b\,b\,b\,c\,c$	35:	$a\,b\,a\,c\,a\,b\,c$	54:	$c\,a\,a\,a\,b\,b\,c$
17:	$a\,b\,a\,b\,a\,c\,c$	36:	$b\,a\,a\,c\,a\,b\,c$	55:	$c\,a\,a\,b\,a\,b\,c$
18:	$b\,a\,a\,b\,a\,c\,c$	37:	$a\,a\,c\,b\,b\,a\,c$	56:	$c\,a\,b\,a\,a\,b\,c$
19:	$a\,a\,a\,b\,b\,c\,c$	38:	$a\,b\,c\,a\,b\,a\,c$	57:	$c\,b\,a\,a\,a\,b\,c$

C Proof of Proposition 6

The proof shall go in two stages: the first part proves Eq. (1), with coefficients a_i being independent of (n,k). The second part, which essentially involves the property $c_{n,n} = c_{n,n-1}$, will deal with the explicit form for $\{a_i\}$.

Let us suppose by induction hypothesis that the expressions corresponding to $c_{n-1,k}$ and $c_{n,k-1}$ hold true:

$$\begin{aligned} c_{n-1,k} &= \sum_{i=0}^{k} \frac{(2n+k+i-3)!!}{i!}\, a_{k-i} = (2n+k-3)!!\, a_k + \sum_{i=1}^{k} \frac{(2n+k+i-3)!!}{i!}\, a_{k-i}, \\ c_{n,k-1} &= \sum_{i=0}^{k-1} \frac{(2n+k+i-2)!!}{i!}\, a_{k-i-1} = \sum_{i=1}^{k} \frac{(2n+k+i-3)!!}{(i-1)!}\, a_{k-i}. \end{aligned}$$

From recursive relation (3), we have

$$\begin{aligned} c_{n,k-1} + (2n+k-1)c_{n-1,k} &= (2n+k-1)!!\, a_k + \sum_{i=1}^{k} \Big[i + (2n+k-1)\Big] \frac{(2n+k+i-3)!!}{i!}\, a_{k-i} \\ &= (2n+k-1)!!\, a_k + \sum_{i=1}^{k} \frac{(2n+k+i-1)!!}{i!}\, a_{k-i} = c_{n,k}\,. \end{aligned}$$

In this fashion, we prove that the aforementioned $c_{n,k}$ comply with relation (7). Regarding coefficients a_i, relation (8) is obtained from the property $c_{n,n} = c_{n,n-1}$ as follows:

$$\begin{aligned} c_{n,n} &= \sum_{i=0}^{n-1} \frac{(4n-i-1)!!}{(n-i)!}\, a_i + (3n-1)!!\, a_n, \\ c_{n,n-1} &= \sum_{i=0}^{n-1} \frac{(4n-i-3)!!}{(n-i-1)!}\, a_i. \end{aligned}$$

Equating the two previous expressions

$$(3n-1)!!\,a_n = -\sum_{i=0}^{n-1}\Big[4n-i-1-(n-i)\Big]\,\frac{(4n-i-3)!!}{(n-i)!}\,a_i,$$

we obtain a_n as

$$a_n = \frac{-1}{(3n-3)!!}\sum_{i=0}^{n-1}\frac{(4n-i-3)!!}{(n-i)!}\,a_i = \frac{-1}{(3n-3)!!}\sum_{j=1}^{n}(3n-3+j)!!\,\frac{a_{n-j}}{j!}\,.$$

D Solution of the first order difference equation

To determine the solution of the recurrence $y_{m+1} = a_m\, y_m + b_m$, with $a_m \neq 0$, divide each term by the following common product

$$\frac{1}{\prod_{i=0}^{m} a_i}\,y_{m+1} - \frac{a_m}{\prod_{i=0}^{m} a_i}\,y_m = \frac{b_m}{\prod_{i=0}^{m} a_i}\,,$$

that is

$$\frac{y_{m+1}}{\prod_{i=0}^{m} a_i} - \frac{y_m}{\prod_{i=0}^{m-1} a_i} = \frac{b_m}{\prod_{i=0}^{m} a_i}\,. \tag{D1}$$

Define now the variable

$$A_k \equiv \frac{y_k}{\prod_{i=0}^{k-1} a_i}\,. \tag{D2}$$

In terms of the new variable Eq. (D1) reads

$$A_{m+1} - A_m = \frac{b_m}{\prod_{i=0}^{m} a_i}\,.$$

Finally perform the telescopic sum

$$\sum_{k=\alpha}^{m}\Big(A_{k+1} - A_k\Big) = A_{m+1} - A_\alpha = \sum_{k=\alpha}^{m}\frac{b_k}{\prod_{i=0}^{k} a_i}\,.$$

Only remains to isolate y_{m+1} in the definition (D2)

$$y_{m+1} = \prod_{i=0}^{m} a_i\,\left[A_\alpha + \sum_{k=\alpha}^{m}\frac{b_k}{\prod_{i=0}^{k} a_i}\right]\,.$$

E Proof of the asymptotic formula, Proposition 7

To derive expression (9) it is enough to consider the asymptotic expansion of the double factorial function appearing in the numerator of Eq. (7) for n large. The key point here is that the part of the argument that involves the summation index i generates a $n^{-i/2}$ factor. Thus, for big n only the first terms in the series a_i contribute to the sum.

The asymptotic behavior of the double factorial can be found in the literature (see, for instance, `http://functions.wolfram. com/06.02.06.0008.01`). For integer m, it reads as:

$$m!! \;=\; \sqrt{2}\left[\sqrt{\frac{\pi}{2}}\,\right]^{\frac{1+(-1)^m}{2}} m^{\frac{m+1}{2}}\,e^{-\frac{m}{2}}\left(1+\frac{1}{6\,m}+\frac{1}{72\,m^2}-\frac{139}{6480\,m^3}-\frac{571}{155520\,m^4}+\mathcal{O}\left(m^{-5}\right)\right)$$

We now make the substitution $m \to 2n+2k-i-1$. In order to obtain a power series for big n it suffices to develop $\ln(1+x)$ and e^{-x} as Taylor series. With the definitions

$$\alpha \equiv k \,-\, i/2 \qquad \text{and} \qquad \beta \equiv \frac{2\alpha-1}{8n}, \tag{E1}$$

we now obtain:

$$(2n+2\alpha-1)^{n+\alpha} = (2n)^{n+\alpha} e^{\alpha-1/2} \Bigg(1 + (2\alpha+1)\beta + \frac{1}{6}\,(12\alpha^2-4\alpha-13)\beta^2$$

$$+ \frac{1}{6}\,(4\alpha^2-4\alpha-11)(2\alpha-3)\beta^3 + \frac{1}{360}\,(240\alpha^4-1440\alpha^3+1640\alpha^2+3352\alpha-5521)\beta^4 + \mathcal{O}\left(n^{-5}\right)\Bigg) . \tag{E2}$$

As far as the sum of powers is concerned, $S \equiv \left(1+\frac{1}{6\,m}+\frac{1}{72\,m^2}-\ldots\right)$, defining $\gamma \equiv \frac{1}{2n}$, we have

$$S = 1 + \frac{1}{6}\gamma - \frac{1}{3}\left(\alpha - \frac{13}{24}\right)\gamma^2 + \frac{1}{18}\left(12\alpha^2 - 13\alpha + \frac{1121}{360}\right)\gamma^3$$

$$- \frac{1}{1080}\left(1440\alpha^3 - 2340\alpha^2 + 1121\alpha - \frac{21821}{144}\right)\gamma^4 + \mathcal{O}\left(n^{-5}\right) . \tag{E3}$$

Collecting all contributions, we get

$$(2n+2k-i-1)!! \;=\; \sqrt{2}\left[\sqrt{\frac{\pi}{2}}\,\right]^{\frac{1-(-1)^i}{2}} e^{-n}\,(2n)^{n+k-i/2}\times$$

$$\left(1 + \left(\alpha^2 - \frac{1}{12}\right)\gamma + \frac{1}{12}\left(6\alpha^4 - 8\alpha^3 - \alpha^2 + 2\alpha + \frac{1}{24}\right)\gamma^2\right.$$

$$\left. + \frac{1}{288}\left(48\alpha^6 - 192\alpha^5 + 180\alpha^4 + 64\alpha^3 - 95\alpha^2 - 4\alpha + \frac{1003}{180}\right)\gamma^3 + \mathcal{O}\left(n^{-4}\right)\right) . \tag{E4}$$

To obtain the asymptotic expression (11) we need to add up the first $i = 0\ldots 4$ terms of the sum (9), apply the just obtained asymptotic series (E4) and group the terms in powers of n. In order to get a simple formula we also applied the following equality:

$$\frac{(2n)^{-i/2}}{(k-i)!} = \frac{1}{k!} \times \frac{k(k-1)\cdots(k+1-i)}{(2n)^{i/2}} .$$

F Generator of general classes of words $\mathcal{C}_{n,k}$

Next we provide an algorithm, based on the recurrence schema described in the subsection 3.1 of the main text, that sequentially generates, with low memory requirements, all words in $\mathcal{C}_{n,k}$.

Algorithm A (*General Words Generator*): Given numbers n and k, the algorithm visits all words $w = c_1c_2\ldots c_{2n+k}$ in $\mathcal{C}_{n,k}$ starting by the word $1122\ldots nn123\ldots k$. Variable j is the name the letter (number), placed at position p, we wish to move. An auxiliary vector $a_1a_2\cdots a_n$ is used to track the number of available interchanges.

A1. [**Initialize.**] Set $c_{2i-1} \leftarrow i$ and $c_{2i} \leftarrow i$ for $1 \le i \le n$, also set $c_{2n+i} \leftarrow i$ for $1 \le i \le k$.
Set $a_i \leftarrow 0$ for $1 \le i < n$ and set $a_n \leftarrow \min(k, n-1)$.

A2. [**Visit.**] Visit the word $w = c_1c_2\ldots c_m$ and set $j \leftarrow 2$.

A3. [**Try to move left.**] Find least p such that $c_p = j$. Find greatest $q < p$ such that $c_q < j$. If q is found set $c_p \leftarrow c_q$ and $c_q \leftarrow j$. Also set $a_i \leftarrow 0$ for $1 \le i < j-1$ and go to A2.

A4. [**Try to move right.**] Find least $q > p$ such that $c_q = j$. Find least $s > q$ such that $c_s < j$ ($s < n+q$). If found and $c_s \le a_j$ then set $c_p \leftarrow c_s$, $c_s \leftarrow j$ and actualize the auxiliary vector: set $a_{j-1} \leftarrow \min(j-1, a_{j-1}+1)$ and $a_i \leftarrow 0$ for $1 \le i < j-1$ and go to A6.

A5. [**Increase j.**] Set $j \leftarrow j+1$. Terminate if $j > n$, otherwise set *out* $\leftarrow$ *false*.

A6. [**Prepare to actualize.**] Set $\ell \leftarrow j-1$ and $r \leftarrow a_\ell$. Also set $u \leftarrow \min(\ell, r)$, $t \leftarrow 1$, $s \leftarrow 0$ and $p \leftarrow 1$.

A7. [**Actualize.**] Repeat $p \leftarrow p+1$ until $c_p \leq \ell$. If $t \leq \ell$ set $c_p \leftarrow t$, otherwise set $c_p \leftarrow t-\ell$. If $s = 1$ and $r = \ell + u$ we are done; go to A8. If $s = 1$ then set $t \leftarrow t + 1$. If $r \leq \ell$ set $s \leftarrow 1 - s$. Return to A7.

A8. [**Visit?**] If *out* = *true* then go to A2, otherwise set *out* $\leftarrow$ *true* and go to A3.
